# Editorial: Epigenetic and gene regulation underlying crosstalk in plant development and stress responses

**DOI:** 10.3389/fgene.2024.1427102

**Published:** 2024-07-18

**Authors:** Romit Seth, Massimo Iorizzo, Tony Kipkoech Maritim, Ram Kumar Sharma

**Affiliations:** ^1^ Plants for Human Health Institute, North Carolina State University, Kannapolis, NC, United States; ^2^ Department of Horticultural Science, North Carolina State University, Raleigh, NC, United States; ^3^ Tea Breeding and Genetic Improvement Division, KALRO-Tea Research Institute, Kericho, Kenya; ^4^ Biotechnology Department, CSIR-Institute of Himalayan Bioresource Technology (CSIR-IHBT), Palampur, Himachal Pradesh, India; ^5^ Academy of Scientific and Innovative Research (AcSIR), Ghaziabad, India

**Keywords:** plant-environment interactions, epigenetic, transcriptomics (RNA sequencing), epigenetics (chromatin remodelling), genomics

Understanding the intricate interactions between plants and their environment is paramount in modern genetics. Plants, inherently sessile beings, harbour complex genomes and evolved sensory and signalling mechanisms to perceive and react to biotic and abiotic stresses. Their ability to navigate stresses such as drought, temperature fluctuations, salinity, pathogens, and wind gusts is not only vital for survival but also for maintaining productivity. To cope with these challenges, plants have developed sophisticated mechanisms to record the consequences of past encounters, facilitating quicker and more efficient responses upon subsequent exposure to the same stressors.

The recent Research Topic “Epigenetic and gene regulation underlying crosstalk in plant development and stress responses” delved into this fascinating arena, featuring one data report and four original research articles elucidating the molecular genetics and epigenetic responses in plants to various environmental cues. The overarching goal was to uncover the epigenetic and transcriptomic intricacies that underlie the complex traits associated with plant-environment interaction.

In a study by Wu et al., the critical role of the AGC protein kinases in sexual reproduction and abiotic stress response in *Brassica rapa* was studied through RNA Seq expression analysis. The authors identified 62 *Br*AGC genes distributed across the *B. rapa* genome. The position of these genes was highly colinear with *Arabidopsis thaliana At*AGC protein kinases. The research unveiled preferential expression of *Br*AGCs in flowers, and upregulation in response to different pollination types. Three genes *Br*AGC26, *Br*AGC33, and *Br*AGC44 stood out for their potential role in regulating pollen-pistil interaction and responding to abiotic stress. In summary, the study reveals the complex genetic pathways influencing reproductive processes and stress tolerance in *B. rapa*, offering insights into the molecular mechanisms essential for these plant functions. It emphasizes the importance of understanding gene families like *Br*AGC in helping plants manage reproductive success amidst environmental challenges.

Similarly, Wang et al., studied the impact of exogenous Methyl Jasmonate application on *Artemisia argyi* transcriptome, revealing the activation of calmodulin CaM4 and auxin response factor (ARF) in the MAPK signalling pathway. This study unveiled the complex signalling cascades triggered by plant hormones and their crosstalk with defense responses, particularly in the context of plant-pathogen interactions. By elucidating the roles of key signalling molecules like CaM4 and ARF, researchers gain a deeper understanding of how plants perceive and respond to external stressors.

Another study by Shi and Du shaded light on the molecular mechanisms underlying drought resistance in tomatoes. Identification of differentially expressed genes and their responses to exogenous ABA and Ca^2+^ treatments provided a solid foundation for further research on the role of *Sl*CNGC genes in enhancing tomato’s resilience to drought stress.

The study by Song et al. unravelled the intricate flowering mechanisms in blueberry plants, emphasizing the pivotal role of FLOWERING LOCUS T (*Vc*FT) and SUPPRESSOR OF OVEREXPRESSION OF CONSTANS 1 (*Vc*SOC1) in regulating floral initiation and bud breaking. By manipulating the *Vc*FT/*Vc*SOC1 ratio, researchers were able to induce precocious flowering and control flower bud formation. These findings offer a hypothesis on blueberry flowering, proposing that the balance between *Vc*FT and *Vc*SOC1 expression levels dictates the timing of flower bud development and blooming. This research provides valuable insights into the molecular pathways governing flowering in deciduous woody crops, paving the way for potential advancements in crop production and breeding strategies. Additionally, this will also improve our understanding of how genetic modifications can impact crucial developmental processes in plants, offering new avenues for enhancing crop productivity and reproductive success in blueberry cultivation.

While genetic alterations are pivotal in plant responses to environmental stresses, the complexity of plant-environment interactions extends beyond genetic mechanisms alone. Epigenetic regulation plays a fundamental role in transcriptional priming, protein conformation, and the modulation of hormonal and metabolite profiles in plants. Understanding these epigenetic nuances is crucial for unravelling the molecular intricacies of plant-environment crosstalk.

*Papaver somniferum* (opium poppy), a medicinal plant, has been used for centuries for its diverse alkaloids with pharmacological properties. Jia et al., provided a data report predicting the cis-regulatory elements in six tissues, shedding light on regulatory networks. They used transposase accessible chromatin sequencing (ATAC-seq) and RNA-Seq data from capsule, stem, fine root, tap root, leaf, and petal tissues to predict open chromatin regions and transcription factor binding sites at a tissue-specific level. The key finding of the study included elucidation of the role of TF HB6 in co-regulating 17 biosynthetic genes and the formation of a gene cluster by two biosynthetic genes. This research offers valuable insights into plant development and secondary metabolism, guiding future studies to uncover the molecular mechanisms and evolution behind these regulatory processes. Additionally, it may assist in future investigations into the epigenetic regulation of gene expression and the development of novel strategies to manipulate gene expression patterns for improved crop traits and secondary metabolite production in this economically important plant species.

In conclusion, the complexity of plant-environment interactions cannot be solely defined by genetic alterations. Epigenetic studies, coupled with whole exome and genome sequencing, offer a promising avenue to unravel the intricacies of plant-environment crosstalk. Moreover, understanding the epigenetic regulation underlying transcriptional priming and protein conformation will provide valuable insights into the mechanistic background of hormonal and metabolic signatures in horticultural and agricultural crop species. As we continue to delve into this fascinating field, this research will pave the way for a more sustainable and resilient agricultural future.

